# Atypical Lung Metastases From Pancreatic Cancer

**DOI:** 10.1002/rcr2.70524

**Published:** 2026-02-18

**Authors:** Ryoju Sato, Yasushi Fukuda, Tadashi Ishida

**Affiliations:** ^1^ Kurashiki Central Hospital Respiratory Medicine Kurashiki Japan

**Keywords:** consolidation, ground‐glass opacity, lepidic, lung metastases, pancreatic cancer

## Abstract

Pulmonary metastases from pancreatic cancer rarely show an alveolar pattern on computed tomography (CT). A 77‐year‐old man presented with a productive cough; CT showed ground‐glass opacities and consolidations, and a tumour in the pancreas was incidentally detected. Biopsy led to a diagnosis of pancreatic cancer with lung metastases.

## Graphical abstract

A 77‐year‐old man presented with a productive cough. CT showed ground‐glass opacities and consolidations. A tumour in the pancreas was incidentally detected on CT, and biopsy led to a diagnosis of pancreatic cancer with lung metastases.

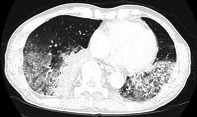

A 77‐year‐old man presented with a wet cough that had persisted for 3 weeks. Chest radiographs and chest computed tomography (CT) showed ground‐glass opacities and consolidations in both lung fields (Figure 1 A,B). Bronchoalveolar lavage showed an elevated lymphocyte percentage of 41% in the cell fraction, with negative cytology. Initially, interstitial lung disease was considered, but a pancreatic mass was incidentally detected on CT (Figure 1 C). A cryobiopsy of the lung and an endoscopic ultrasound‐guided fine needle aspiration (EUS‐FNA) of the pancreatic mass were performed. The diagnosis was lung metastases from pancreatic intraductal papillary mucinous carcinoma (Figure 2). Chemotherapy was performed, but the patient died 3 months after diagnosis.

**FIGURE 1:**
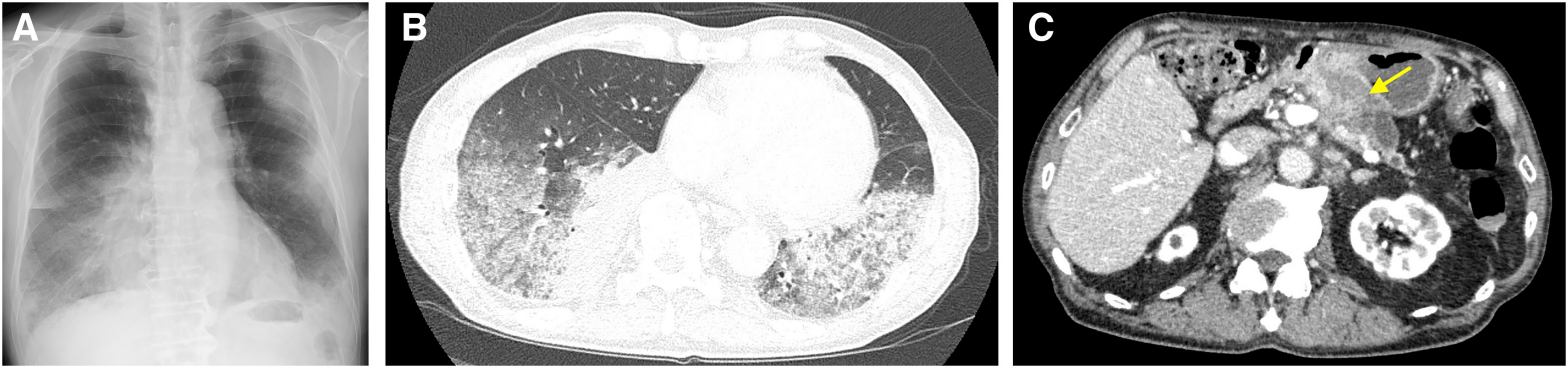
(A) Chest radiograph showing ground‐glass opacities and consolidations in both lung fields. (B) CT showing ground‐glass opacities and consolidations in both lung fields. (C) Contrast‐enhanced CT showing a pancreatic mass (an arrow).

**FIGURE 2:**
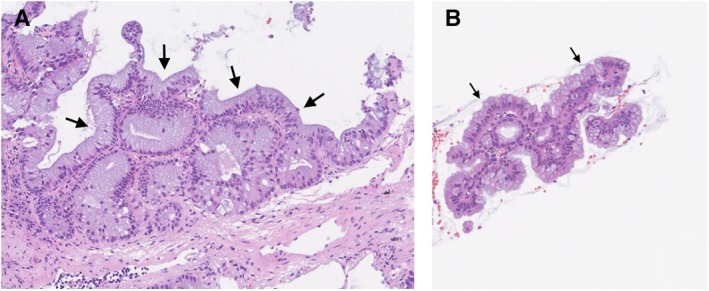
(A) Lung histopathology showing lepidic growth of the metastatic cells from pancreatic intraductal papillary mucinous carcinoma (arrows). (B) Pancreas histopathology showing pancreatic intraductal papillary mucinous carcinoma (arrows).

Pulmonary metastases from pancreatic cancer are typically hematogenous, appearing as multiple, rounded, solid nodules on CT. However, 15%–22% of pulmonary metastases from pancreatic cancer showed an alveolar pattern on CT, and ground‐glass nodules and consolidations were each observed in 4% of cases [1, 2]. The air‐space radiological pattern corresponds on histology to lepidic tumour growth along alveolar walls [1]. In this case, the diagnosis was difficult due to ground‐glass opacities and consolidations mimicking interstitial lung disease. However, lung metastases from pancreatic cancer can appear as various types of CT findings.

## Author Contributions

Ryoju Sato served as the attending physician for this patient and wrote this manuscript. Yasushi Fukuda and Tadashi Ishida supervised the patient's care and revised this manuscript.

## Consent

The authors declare that written, informed consent was obtained for the publication of this manuscript and accompanying images and attest that the form used to obtain consent from the patient complies with the Journal requirements as outlined in the author guidelines.

## Conflicts of Interest

The authors declare no conflicts of interest.

## Data Availability

The data that support the findings of this study are available on request from the corresponding author. The data are not publicly available due to privacy or ethical restrictions.
